# Histogenesis-specific expression of fibroblast activation protein and dipeptidylpeptidase-IV in human bone and soft tissue tumours

**DOI:** 10.1111/j.1365-2559.2009.03399.x

**Published:** 2009-10

**Authors:** Osamu Dohi, Haruo Ohtani, Masahito Hatori, Elichi Sato, Masami Hosaka, Hiroshi Nagura, Eiji Itoi, Shoichi Kokubun

**Affiliations:** 1 Departments of Orthopaedic Surgery, Tohoku University Graduate School of MedicineMito, Japan; 2 Departments of Pathology, Tohoku University Graduate School of MedicineMito, Japan; 3 Department of Orthopaedic Surgery, Tohoku Kosai Hospital, SendaiMito, Japan; 4 Department of Pathology, Mito Medical Centre, National Hospital OrganizationMito, Japan

**Keywords:** bone and soft tissue tumours, dipeptidylpeptidase-IV, fibroblast activation protein, histogenesis, human

## Abstract

**Aims:**

Fibroblast activation protein (FAP)/seprase and dipeptidylpeptidase-IV (DPP-IV)/CD26 are serine integral membrane proteases. They are involved in tissue remodelling, cancer invasion and metastases, mechanisms that are controversial. The aim was to identify cell types that express FAP and DPP-IV in human bone and soft tissue tumours, and to determine whether there are any correlations between the expression of FAP and DPP-IV and the malignant potential of tumours.

**Methods and results:**

This study analysed *in situ* expression in 25 malignant and 13 benign human bone and soft tissue tumours. Reverse transcriptase-polymerase chain reaction analyses confirmed mRNA expression of FAP and DPP-IV in all individuals. Immunohistochemistry using pre-fixed frozen sections revealed that FAP was positive in low-grade myofibroblastic sarcoma, the fibroblastic component of osteosarcomas, and malignant fibrous histiocytomas, but negative in Ewing’s sarcomas and rhabdomyosarcomas. DPP-IV showed similar immunohistochemical results. Among benign tumours, non-ossifying fibromas, desmoid tumours and chondroblastomas expressed both FAP and DPP-IV. Giant cells expressed DPP-IV in giant cell tumours.

**Conclusions:**

Our data suggest that FAP and DPP-IV are consistently expressed in bone and soft tissue tumour cells that are histogenetically related to activated fibroblasts and/or myofibroblasts, irrespective of their malignancy. DPP-IV is also expressed in monocyte–macrophage lineage cells.

## Introduction

Fibroblast activation protein (FAP)/seprase is a serine integral membrane proteinase. The molecular weight of FAP is either 95 or 97 kDa as a monomer and 170 kDa as a dimer. The dimer shows gelatinolytic activity.^1^,^2^ FAP is reported as an antigen that is recognized by antibody F19.^3^,^4^ FAP is not expressed in normal fibroblasts or smooth muscle cells in adults, although strongly expressed in activated fibroblasts,^5^–^8^ including stromal fibroblasts in human cancers^9^ and granulation tissue associated with wound healing.^1^ Dipeptidylpeptidase-IV (DPP-IV)/CD26 has a molecular weight of 105 kDa and is also a serine integral membrane proteinase. It is an activation antigen of T lymphocytes. It is diffusely positive in cells of the proximal tubules of the kidney, small intestinal mucosa, bile ducts, sinusoidal lining cells of the spleen, subsets of vascular endothelial cells and fibroblasts.^7^ Since FAP and DPP-IV are both serine proteases, attention has focused on their potential to promote cancer cell invasion and metastasis, although the results of such studies are controversial.^7^,^10^–^13^ Both FAP and DPP-IV are abundantly expressed in the stroma of >90% of breast, colorectal and lung carcinomas.^7^,^14^,^15^ FAP and DPP-IV are also expressed in sarcomas.^7^ However, detailed cell identification has not been performed for these two proteases in sarcomatous tissues. Furthermore, the relationship between the expression of these two proteinases and the malignancy of tumours has not been clarified.

The present study was designed to identify cell types that express FAP and DPP-IV in human bone and soft tissue tumours, and to determine whether there are any correlations between the expression of FAP and DPP-IV and the malignant potential of tumours.

## Materials and methods

Forty-one bone and soft tissue tumours were analysed. All specimens were obtained by open biopsy or surgical resection from patients, none of whom had received radiation or chemotherapy. All tumours were diagnosed on the basis of clinical features, imaging, conventional histopathology and immunohistochemistry of paraffin-embedded sections. There were 26 malignant and 15 benign tumours: 11 osteosarcomas (eight osteoblastic, two fibroblastic, one chondroblastic), four Ewing’s sarcomas, four malignant fibrous histiocytomas (MFH) of pleomorphic type (undifferentiated high-grade pleomorphic sarcoma), two rhabdomyosarcomas, two low-grade myofibroblastic sarcomas,^16^ one adamantinoma, four giant cell tumours, three schwannomas, one non-ossifying fibroma, one osteoid osteoma, one desmoid tumour, one chondroblastoma, one lipoma, and one elastofibroma. Fibrous granulation tissue obtained from four decubitus ulcers were used as positive controls.^7^ Soft tissues without tumour invasion were used as normal controls, which were removed with the sarcomas. Of these, the two cases of low-grade myofibroblastic sarcoma were originally diagnosed as ‘leiomyosarcoma’. The reasons for the change of diagnosis included (i) expression of smooth muscle actin is common to low-grade myofibroblastic sarcoma and leiomyosarcoma, and (ii) the tumour cells in the two cases lacked enhanced cytoplasmic eosinophilia or blunt-ended nuclei, and did not express calponin on immunohistochemistry.^17^,^18^

mRNA analyses were performed on all specimens according to the method described by Katou *et al.*^19^ The primer sequences were as follows: FAP: +5-GCTAACTTTCAAAAACATCTGGAAAAATG-3 (sense) and +5-GTAATATGTTGCTGTGTAAGAGTATCTCC-3 (anti-sense), and DPP-IV: +5-CTAACTGGACTGGTTCAAATGTTGT-3 (sense) and +5-CAGGGCAAGCTGATGTGTTCACATCTC-3 (anti-sense).

For immunohistochemistry, specimens were fixed in periodate–lysine 4% paraformaldehyde for 6–8 h at 4°C. After washing in phosphate-buffered saline, the fixed specimens were frozen. Primary antibodies were mouse monoclonal anti-FAP (clone F19),^9^ rat monoclonal anti-FAP (clone D8)^20^ and mouse monoclonal anti-DPP-IV (anti-CD26) (clone M-A261; Pharmingen, San Diego, CA, USA). Envision Plus was used as the secondary antibody (Dako, Tokyo, Japan). Isotype-matched control mouse monoclonal antibodies (Dako) were used as the negative control.

The US Food and Drug Administration-approved scoring system for HER2 protein was adopted for semiquantification of the localization patterns of FAP and DPP-IV because the immunoreactivity of both proteases along the cell membrane is similar to that of HER2.^21^ In brief: score 0, negative or positive in <10% of tumour cells; score 1+, weakly positive in >10% of tumour cells; score 2+, moderately positive in >10% of tumour cells; and score 3+, strongly positive in >10% of tumour cells.

## Results

### Confirmation of the expression of FAP and DPP-IV in sarcomatous tissue

**Figure 1:**
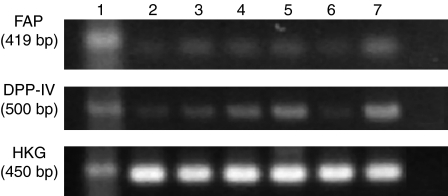
Results of reverse transcriptase-polymerase chain reaction analyses. Lanes: 1, low-grade myofibroblastic sarcoma (case 1); 2, low-grade myofibroblastic sarcoma (case 2); 3, Ewing’s sarcoma; 4, osteosarcoma (a fibroblastic type); 5, osteosarcoma (osteoblastic type); 6, granulation tissue; 7, RNA(−); and HKG, housekeeping gene.

Reverse transcriptase-polymerase chain reaction analyses identified the expression of mRNAs for both FAP and DPP-IV in all specimens examined (Figure 1). Direct sequencing confirmed the reaction products as FAP and DPP-IV by referring to the home page of the National Center for Biotechnology Information (http://www.ncbi.nlm.nih.gov/) (Bethesda, MD, USA).

### Both FAP and DPP-IV are expressed mainly in spindle-shaped cells

**Figure 2:**
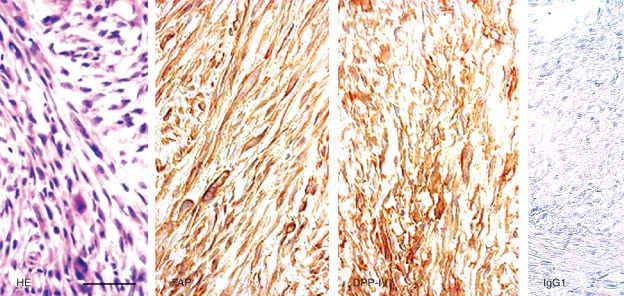
Immunohistochemistry in low-grade myofibroblastic sarcoma, showing strong positivity for both fibroblast activation protein (FAP) and dipeptidylpeptidase-IV (DPP-IV). IgG1, negative control. Scale bar, 50 μm.

**Figure 3:**
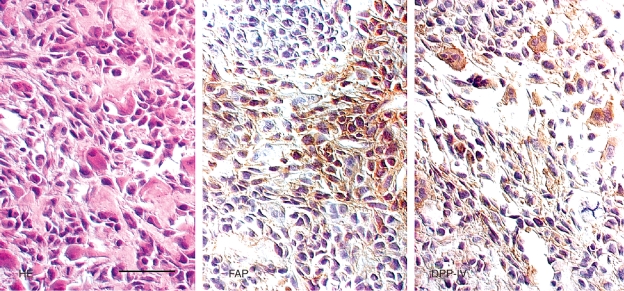
Immunohistochemistry of a fibroblastic area of an osteosarcoma. Fibroblast activation protein (FAP) and dipeptidylpeptidase-IV (DPP-IV) are strongly positive. Scale bar, 25 μm.

**Figure 4:**
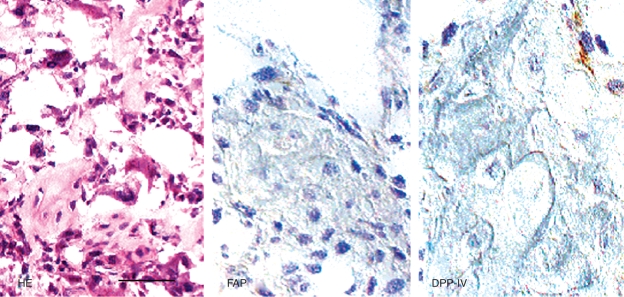
Immunohistochemistry of an osteoblastic area of an osteosarcoma. Fibroblast activation protein (FAP) and dipeptidylpeptidase-IV (DPP-IV) are weakly positive in the osteoblastic area. Scale bar, 25 μm.

**Figure 5:**
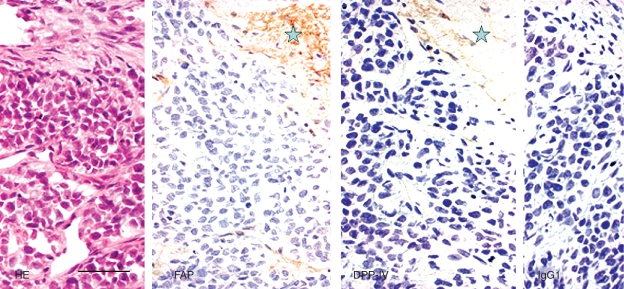
Immunohistochemistry in Ewing’s sarcoma. Tumour cells, small and round in shape, are negative for fibroblast activation protein (FAP) and dipeptidylpeptidase-IV (DPP-IV). Stromal cells in septal areas are positive for FAP and DPP-IV (marked with a star). Scale bar, 50 μm.

To identify the cell types, immunohistochemistry was performed using pre-fixed frozen sections, not conventional paraffin-embedded sections, in order to attain more reliable sensitivity, and the results analysed using the semiquantification method (Table 1). For malignant tumours, we analysed low-grade myofibroblastic sarcoma, osteosarcomas, MFH, rhabdomyosarcoma, Ewing’s sarcoma, and adamantinoma. FAP and DPP-IV were strongly positive in both cases of low-grade myofibroblastic sarcoma, and the positive cells were uniformly spindle-shaped tumour cells (Figure 2). All of the 11 osteosarcomas also expressed FAP and DPP-IV. As summarized in Table 1, it is notable that positive cells for FAP and DPP-IV were observed specifically in fibroblastic areas (Figure 3), whereas they were sparse in osteoblastic (Figure 4) and chondroblastic areas. The positive cells in fibroblastic areas were spindle-shaped fibroblastic tumour cells. FAP and DPP-IV were also positive in fibroblastic tumour cells in two of four and four of four MFHs, respectively. In contrast, no tumour cells expressed FAP or DPP-IV in four Ewing’s sarcomas, two rhabdomyosarcomas, or one adamantinoma. However, it should be noted that reactive fibroblasts in the fibrous septum of all four Ewing’s sarcomas expressed FAP and DPP-IV moderately to weakly (Figure 5). In addition to fibroblastic cells, DPP-IV was also expressed in osteoclast-like giant cells in five of 11 osteosarcomas.

**Table 1:** Results of immunohistochemistry

			Score	
Diagnosis (number of cases)	Case		FAP	DPP-IV
Low-grade myofibroblastic sarcoma (*N* = 2)	1		3	3
	2		3	2
Osteosarcoma	3	F	2	2
Fibroblastic type (*N* = 2)		O	1	0
		C	0	0
	4	F	2	0
		O	1	0
		C	0	0
Osteoblastic type (*N* = 8)	5	F	2	0
		O	0	0
		C	-	-
	6	F	3	3
		O	1	1
		C	-	-
	7	F	3	3
		O	1	0
		C	-	-
	8	F	2	2
		O	0	1
		C	0	0
	9	F	1	2
		O	0	1
		C	0	0
	10	F	2	3
		O	0	0
		C	0	0
	11	F	1	3
		O	0	0
		C	-	-
	12	F	2	3
		O	0	0
		C	0	0
Chondroblastic type (*N* = 1)	13	F	1	2
		O	0	0
		C	0	0
Malignant fibrous histiocytoma (*N* = 4)	14		0	3
	15		0	3
	16		3	3
	17		2	3
Rhabdomyosarcoma (*N* = 4)	18		0	0
	19		0	0
Ewing’s sarcoma (*N* = 4)	20		0	0
	21		0	0
	22		0	0
	23		0	0
Adamantinoma (*N* = 1)	24		0	0
Non-ossifying fibroma (*N* = 1)	26		3	3
Giant cell tumor (*N* = 4)	27	G	1	3
		M	2	1
		F	2	1
	28	G	1	3
		M	1	2
		F	2	1
	29	G	3	3
		M	0	3
		F	1	2
	30	G	1	3
		M	1	3
		F	2	2
Desmoid (*N* = 1)	31		2	3
Chondroblastoma (*N* = 1)	32		2	2
Schwannoma (*N* = 3)	33		0	1
	34		0	0
	35		1	0
Lipoma (*N* = 1)	36		0	0
Elastofibroma (*N* = 1)	37		0	0
Osteoid osteoma (*N* = 1)	38		3	3
Granulation tissues			3	3

Evaluation method for HER2 immunoreactivity was adopted for semiquantification. Pleomorphic malignant fibrous histiocytoma is also referred to as ‘undifferentiated pleomorphic high-grade sarcoma’.

F, fibroblastic area; O, osteoblastic area; C, chondroblastic area; G, giant cells; M, mononuclear cells; FAP, fibroblast activation protein; DPP-IV, dipeptidylpeptidase-IV.

**Figure 6:**
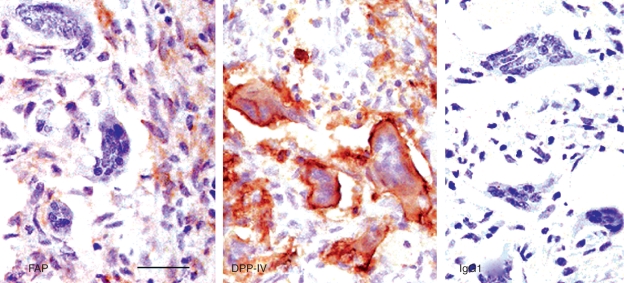
Immunohistochemistry of a giant cell tumour. Fibroblast activation protein (FAP) is expressed in spindle-shaped fibroblastic cells, whereas dipeptidylpeptidase-IV (DPP-IV) is positive in giant cells. Scale bar, 25 μm.

**Figure 7:**
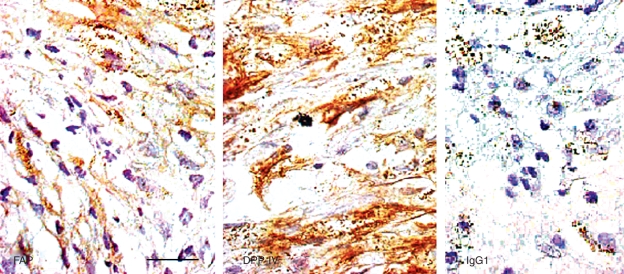
Immunohistochemistry of a non-ossifying fibroma. Spindle-shaped tumour cells express both fibroblast activation protein (FAP) and dipeptidylpeptidase-IV. Scale bar, 25 μm.

We then analysed benign tumours to confirm the expression of FAP and DPP-IV. To analyse giant cell tumours, we subdivided the cellular components into giant cells, mononuclear round cells, and spindle-shaped fibroblastic cells. As summarized in Table 1, all these components of giant cell tumours expressed FAP and DPP-IV (Figure 6). FAP and DPP-IV were also positive in spindle-shaped tumour cells of one non-ossifying fibroma (Figure 7), one chondroblastoma, and one desmoid tumour. Osteoclast-like giant cells were positive for FAP and DPP-IV in one osteoid osteoma. Spindle cells expressed FAP and DPP-IV in one of three schwannomas. No tumour cells expressed FAP or DPP-IV in lipoma or elastofibroma.

## Discussion

The present study has clearly shown that fibroblast-like spindle tumour cells strongly express FAP and DPP-IV in low-grade myofibroblastic sarcoma, fibroblastic areas of osteosarcoma, MFH, non-ossifying fibroma, desmoid tumour, and chondroblastoma. The expression pattern is similar to that of activated fibroblasts and/or myofibroblasts in granulation tissue. In contrast, neither FAP nor DPP-IV is expressed in round or oval cells in Ewing’s sarcoma or rhabdomyosarcoma.

Our results demonstrate the following two points. First, expression of FAP and DPP-IV is clearly independent of the malignant potential of the tumours. Second, it is rather related to the histogenesis of the tumour cells, i.e. positive tumour cells are related to activated fibroblasts and/or myofibroblasts. Myofibroblasts are activated fibroblasts that have some of the characteristics of smooth muscle cells, such as contractile properties.^22^ Eyden has described how tumour cells with complete myofibroblastic differentiation show immunoreactivity for smooth muscle actin, prominent rough endoplasmic reticulum, peripheral smooth-muscle type myofilaments, and fibronexus junctions. It is reasonable therefore that low-grade myofibroblastic sarcomas and fibroblastic areas of osteosarcoma abundantly expressed FAP and DPP-IV. The positivity for FAP and DPP-IV in MFH could be related to the previous demonstration of myofibroblastic differentiation in storiform–pleomorphic MFH.^23^,^24^ In sharp contrast, negative tumours in the present study showed other differentiation phenotypes. Chondroblastic and osteoblastic areas of osteosarcoma, both apparently showing different phenotypes, showed weak expression of FAP and DPP-IV. No expression was observed in Ewing’s sarcoma or rhabdomyosarcoma, and the two sarcomas are thought to be derived from a different cell lineage: Ewing’s sarcoma is considered to be of neuroectodermal origin^25^ and rhabdomyosarcoma of striated muscle origin. Therefore, our data indicate that FAP and DPP-IV are positive in an activated fibroblast/myofibroblast-like lineage, and negative in other cell lineages, including osteoblastic cells (Table 1).

We then considered the relationship to the malignant potential of the tumours, given that both FAP and DPP-IV are serine integral membrane proteinases. This suggests their involvement in cancer invasion and metastasis. However, as shown below, the situation is rather complicated. Cheng *et al.*^13^ indicated that FAP is a tumour promoter, whereas Ramirez-Montagut *et al.*^12^ have described FAP as a tumour suppressor. Ariga *et al.*^8^ reported that the degree of FAP expression in breast cancer stromal cells was associated with a longer survival of patients, and they suggested that FAP may be involved in tissue remodelling of the cancer stroma. The situation for DPP-IV is similar. On the one hand, DPP-IV has been reported to play important roles in metastasis of cancer cells.^14^ DPP-IV is involved with endothelial cell functions as an adhesion receptor for fibronectin on cancer cells.^20^ The expression of fibronectin correlates with the ability of cancer cells to bind to DPP-IV and metastasize to the lung.^20^ The ability of breast cancer cells to capture fibronectin molecules suggests that DPP-IV–fibronectin binding may be a relatively common mechanism necessary for lung metastasis.^20^ On the other hand, DPP-IV may act as a tumour suppressor by regulating levels of E-cadherin in cells through an unknown mechanism.^15^ Overall, the significance of FAP and DPP-IV in cancer invasion and metastasis is controversial. Our results indicate that both malignant and benign tumours express FAP and DPP-IV. Therefore, the expression of FAP and DPP-IV is irrelevant to the malignant potential of the tumour as far as bone and soft tissue tumours are concerned.

In addition to the above-mentioned expression in fibroblastic cells, DPP-IV is associated with another notable result of this study. Giant cells in giant cell tumours of bone were positive for DPP-IV. Giant cell tumours were formerly named osteoclastomas, and there has been debate concerning the relationship between giant cells in giant cell tumours and osteoclasts. Osteoclasts in normal bone tissue resemble macrophages in their histogenesis and function,^26^ and they are positive for DPP-IV.^18^ Therefore, it is probable that in addition to fibroblastic cells, DPP-IV is also positive in cells of the monocyte–macrophage system. DPP-IV expression in reactive giant cells of osteosarcoma further supports this notion.

In conclusion, both FAP and DPP-IV are expressed in tumour cells that are histogenetically related to activated fibroblasts and/or myofibroblasts, and their expression is not dependent on the malignancy of tumours. Our data suggest that FAP and DPP-IV can be used as markers of activated fibroblasts and/or myofibroblasts. DPP-IV may also be used as a marker of cells in the monocyte–macrophage lineage.

## Abbreviations:

DPP-IV: dipeptidylpeptidase-IV
FAP: fibroblast activation protein
MFH: malignant fibrous histiocytoma
